# Biological Clock and Inflammatory Bowel Disease Review: From the Standpoint of the Intestinal Barrier

**DOI:** 10.1155/2022/2939921

**Published:** 2022-03-14

**Authors:** Yonggang Tian, Dekui Zhang

**Affiliations:** Department of Gastroenterology, Lanzhou University Second Hospital, Lanzhou, Gansu Province, China

## Abstract

Inflammatory bowel disease is a group of chronic, recurrent, nonspecific inflammatory diseases of the intestine that severely affect the quality of life of patients. The pathogenesis of this disease is caused by complex and interactive neural networks composed of factors such as genetic susceptibility, external environment, immune disorders, and intestinal barrier dysfunction. It is well known that there is a strong link between environmental stressors (also known as circadian clocks) that can influence circadian changes and inflammatory bowel disease. Among them, the biological clock is involved in the pathogenesis of inflammatory bowel disease by affecting the function of the intestinal barrier. Therefore, this review is aimed at systematically summarizing the latest research progress on the role of the circadian clock in the pathogenesis of inflammatory bowel disease by affecting intestinal barrier functions (intestinal mechanical barrier, intestinal immune barrier, intestinal microecological barrier, and intestinal chemical barrier) and the potential clinical value of clock genes in the management of inflammatory bowel disease, for the application of circadian clock therapy in the management of inflammatory bowel disease and then the benefit to the majority of patients.

## 1. Introduction

Inflammatory bowel disease (IBD) is a group of chronic, nonspecific, recurrent inflammatory diseases caused by genetic [1], environmental [2], immune [3], intestinal microecology [4, 5], and other factors [6]. It mainly includes two subtypes of ulcerative colitis (UC) and Crohn's disease (CD) [7]. The disease not only affects the gastrointestinal system but also has a wide range of extraintestinal manifestations such as the eyes [8], skin [9], and joints [10]. In addition, patients with IBD have an increased risk of cardiovascular disease [11], Parkinson's disease [12], cerebrovascular disease [13], diabetes [14], psychosis, and suicide [14], in which all seriously affect the quality of life of patients and cause a huge economic burden. IBD is a 21^st^-century global disease [15]. There are approximately 3 million patients with IBD in the United States and Europe, and the prevalence of IBD is estimated to exceed 0.3% in many countries in North America, Oceania, and Europe, but the incidence is stable or declining in North America and Europe and rising in the newly industrialized countries [16]. In addition, the study believes that by 2020, the incidence of IBD in newly industrialized countries will be accelerated [17]. This means that the management of IBD in various countries will face a severe situation in the future.

Although many factors are involved in the pathogenesis of IBD, intestinal barrier dysfunction is one of the key links in the pathogenesis of IBD [18]. Previous studies have identified defects in many specific components of the gut barrier in IBD patients, ranging from the composition of the mucus layer to adhesion molecules that regulate paracellular permeability, and these alterations contribute to the persistence of chronic mucosal inflammation [19], which is also one of the root causes of persistent IBD disease. Another study found that intestinal barrier dysfunction was several years earlier than the clinical diagnosis of IBD, which led to new thinking to prevent the occurrence of IBD through early intervention of intestinal barrier function [20].

Based on the above-mentioned new strategies to prevent the occurrence of IBD by maintaining the homeostasis of the intestinal barrier at an early stage, it is the primary task to actively search for the key factors that can lead to the disturbance of the intestinal barrier. Studies suggest that pathogens, xenobiotics, and food can disrupt the intestinal barrier, promote systemic inflammation and tissue damage, and even lead to other diseases including IBD [21]. It is well known that the biological clock is an intrinsic adaptive mechanism of the human body, which plays an important role in maintaining human body health and disease activities [22]; however, due to the rapid development rhythm and the high pressure of life in modern society, the disturbance of biological clock has become a common feature of modern society [23]. More and more studies have found that there is a certain relationship between abnormal circadian rhythm and IBD [24–30]. Among them, part of the reason is that the biological clock can affect the intestinal barrier function and participate in the pathogenic link of IBD [23, 31, 32]. Therefore, this paper reviews the potential role of the circadian clock in the pathogenesis of IBD and its clinical application value from the perspective of affecting the intestinal barrier function of IBD, to better utilize the principle of the circadian clock to manage IBD, and then benefit IBD patients.

## 2. The Composition and Function of the Intestinal Barrier

**Figure 1:**
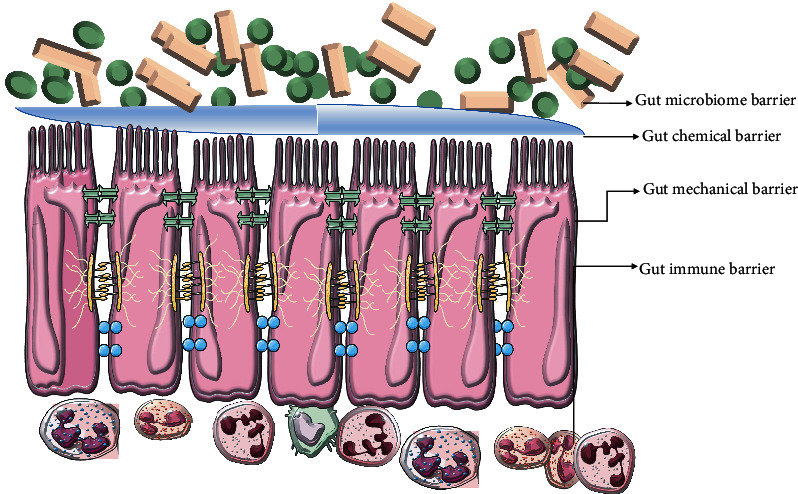
Compositional pattern of four gut barriers.

The intestinal barrier is a highly complex “precise instrument” that interacts with the body's daily intake of a large number of nutrients and various pathogenic microorganisms. It is composed of the intestinal mechanical barrier, intestinal immune barrier, intestinal microecological barrier, and intestinal chemical barrier (Figure 1). These four barriers interact and together constitute a complex interactive neural network to maintain human intestinal health and repair.

### 2.1. The Intestinal Mechanical Barrier

The intestinal mechanical barrier is the structural basis for epithelial selective permeability and barrier function. It is well known that the intestinal mechanical barrier is composed of intestinal mucosal cells and intercellular junctions [21]. Among them, epithelial cells are composed of absorptive cells, goblet cells, and a small number of endocrine cells, which are monolayer columnar. It should be noted that the small intestine also has Paneth cells and undifferentiated cells, which are always in constant renewal, which can ensure their absorption of nutrients, electrolytes, and water; at the same time, it can maintain the effective defense function against various toxins and antigens in the intestinal cavity. Additionally, epithelial cells maintain their selective barrier function by forming complex protein-protein networks that mechanically connect adjacent cells and seal intercellular spaces. The protein network connecting epithelial cells forms 3 adhesion complexes are as follows: tight junctions, desmosomes, and adherens junctions. These complexes consist of transmembrane proteins that interact with neighboring cells extracellularly and with adaptor proteins attached to the cytoskeleton inside the cell; the mechanical barrier is the largest and most important barrier against various pathogenic microorganisms in the gut [33]. Among them, studies have found that tight junctions are dynamic structures composed of apical multiprotein complexes, including tetraspanins of the claudin family and occludin-related Marvel domain proteins, and junctional adhesion molecules. Dense “patches” of scaffolding molecules are anchored to transmembrane proteins, including occlusive bands, which are directly linked to the intracellular cytoskeleton (actin and microtubules) and regulatory proteins such as aPKC, G proteins, Rab1, and Rab3B connected; strong expression of these molecules reduces paracellular permeability, thus limiting the possibility of passage of bacteria and substances. In addition, tight junctions, as one of the important components of the epithelial barrier, are frequently threatened by proinflammatory mediators, pathogenic viruses, and bacteria [34]. Therefore, tight junctions must be able to respond quickly and coordinate, which requires a complex management system to coordinate the assembly state of the tight junction polyprotein network [30]. In conclusion, tight junctions are important “ramps” regulating epithelial permeability and paracellular diffusion, and their structural and functional defects are the main cause of increased epithelial permeability and paracellular permeability. In addition to this, desmosomes are localized dense plaques linked to keratin filaments that are specialized for strong adhesion, with a strong adherent state, providing mechanical integrity to the intestinal mucosal barrier. Adhesin junctions mediate cell-cell adhesion through the action of connexins and cadherins, which play a key role in epithelial integrity and exhibit remarkable plasticity [33]. In conclusion, impaired intestinal epithelial barrier function allows a variety of toxins, pathogens, symbionts, dietary food components, and other small molecules to enter the deeper layers of the gut from the lumen. Continued invasion of the gut subepithelial lamina propria by any of the above factors results in the recruitment and activation of immune cells, which can lead to inflammation [35].

### 2.2. The Intestinal Immune Barrier

The intestinal immune barrier is the “pioneer warrior” of the intestinal barrier against various pathogenic factors. It maintains intestinal homeostasis by cooperation between distinct immune cell subsets in the epithelium, lamina propria, and gut-associated lymphoid tissue [36]. Among them, the epithelial layer is not only the intestinal mechanical barrier but also the first and most important innate immune barrier, which plays an important role in maintaining immune function. In addition, immune cells located in the lamina propria and gut-associated lymphoid tissue also play important physiological functions in intestinal immunity. Studies have found that natural killer T cells play an important role in host intestinal defense and maintenance of intestinal barrier function by controlling microbial colonization and coordinating the functions of other intestinal cells. Specifically, gut NK T cells sense lipids presented by CD11c+ cells, thereby regulating NKT cell homeostasis and activation. In turn, natural killer T cells (directly and indirectly) regulate the function of other gut immune cells and the composition and stratification of gut bacteria. CD1d-mediated cross talk between natural killer T cells and intestinal epithelial cells regulates IL-10 secretion, while CD1d involvement in group 3 ILC3 induces IL-22 production, both cytokines that contribute to the control of intestinal track's steady state [37]. Intestinal intraepithelial lymphocytes (IELs) are a special group of mucosal T lymphocytes that exhibit high activation thresholds and low reactivity to most antigens from the intestinal lumen to maintain intestinal immune tolerance. In particular, CD8*αα*+ TCR*αβ*+ IELs, TCR*γδ*+ IELs, and CD4+ CD8*αα*+ IELs show great potential in maintaining intestinal immune tolerance and regulating intestinal immunity. However, once the intestinal microenvironment is abnormal or intestinal tolerance is disrupted, intestinal intraepithelial lymphocytes may be abnormally activated and lead to the occurrence of disease [38]. Of course, there are other immune cells such as mast cells and macrophages that secrete inflammatory mediators, activate complement, increase blood flow, dilate capillaries, increase permeability, and deposit fibrin networks, which are involved in the pathogenesis of CD [39].

### 2.3. The Intestinal Microbial Barrier

The intestinal microbial barrier is composed of microbes (including bacteria, fungi, and viruses) and microbial metabolites that live in the gut. The composition of this microbial barrier is host-specific, evolves continuously throughout the life of an individual, and is susceptible to exogenous and endogenous factors. The gut microbiota is particularly relevant to host defense, immune response, metabolic energy intake, and nutrition [30]. Among them, gut microbes are a double-edged sword for human health. Probiotic strains currently in development are an effective form of treatment for inducing remission in patients with mild to moderate UC [40]. However, studies have found that the intestinal flora secretes a variety of metabolites and bacteriocins, and some bacteria also activate the immune system by expressing specific antigens, adhering to the intestinal epithelium, and interacting with pattern recognition receptors, prompting immune cells to secrete large amounts of proinflammatory factors, thereby causing intestinal inflammation [41]. For example, it has been found that lipopolysaccharide, a toxin produced by intestinal bacteria, can induce HEK-TLR4 cells to produce NF-*κ*B and proinflammatory IL-8 in a TLR4-dependent manner, leading to the occurrence of IBD [42]. In addition, short-chain fatty acids can alter chemotaxis and phagocytosis, induce reactive oxygen species production, alter cell proliferation and function, have anti-inflammatory, antitumor, and antibacterial effects, and alter intestinal integrity, all of which suggest that they are essential for maintaining intestinal and major players in immune homeostasis [43].

### 2.4. The Intestinal Chemical Barrier

The intestinal chemical barrier is mainly composed of a mucus layer composed of digestive juice, various digestive enzymes, lysozyme, bile acids, and mucin. The study found that the mucus layer on the surface of luminal epithelial cells is composed of glycosylated mucin polymers produced by goblet cells. A bilayer mucus layer on the epithelial surface protects the host from pathogenic microorganisms and their inflammatory mediators in the gut. The first layer of mucus is approximately 50 mm, which is almost bacteria-free and able to keep the microbiota away from the epithelial barrier and limit inflammation. In the second layer of mucus, the microbiota is present and involved in its degradation, a process that is constantly changing every day [44]. Among them, various substances that make up the intestinal chemical barrier have certain functions. Lysozyme can hydrolyze peptidoglycan in bacterial cell walls to exert antibacterial effect [45], digestive enzymes can decompose macromolecular substances in food into small molecular substances for easy digestion and absorption [46], and bile acids can affect gastrointestinal motility, sensation, secretion, regulation of function, intestinal barrier permeability, and inflammatory responses [47]. In conclusion, the intestinal mucus layer plays a major role in protecting the gut from mechanical, chemical, and biological factors and contributes to the maintenance of intestinal homeostasis [48].

### 2.5. IBD Intestinal Barrier Function

Intestinal barrier dysfunction has emerged as a hallmark event of IBD [49]. Some studies on human subjects have shown that compared with healthy control subjects, IBD patients have reduced fecal and mucosa-associated microbiome diversity, decreased probiotic microorganisms, and increased pathogenic bacteria biota [50, 51], which is undoubtedly a disruption of gut microbial homeostasis. In addition, the various components of the mucosal immune system in IBD include intestinal epithelial cells, innate lymphocytes (macrophages/monocytes, neutrophils, and dendritic cells), adaptive immune cells (T and B cells), and abnormal changes in the mediators (cytokines and chemokines) it secretes; these immune factors may lead to activation of innate immune responses through autophagy, mucosal susceptibility, or defective luminal antigen-antibody binding responses, which may be mediated by enhanced Toll-like receptor activity. Antigen-presenting cells then mediate the differentiation of naive T cells into effector T helper (Th) cells, including Th1, Th2, and Th17, which alter intestinal homeostasis and lead to IBD [52]. Mucin, as the main component of the intestinal chemical barrier, plays an important role in maintaining the intestinal barrier function, but changes in the number of secreted mucins, structural changes in the core of mucin glycoproteins, and mucin oligosaccharides occur in IBD patients. Sulfation and sialylation of residues are associated with reduced mucus barrier function [53].

## 3. Molecular Mechanism of the Biological Clock

The biological clock is habitually called circadian rhythm, which is an inherent adaptive mechanism in the process of biological evolution. From most single-celled organisms to humans, there is a 24-hour biological rhythm pattern, which plays an important role in maintaining human health

### 3.1. Effect

**Figure 2:**
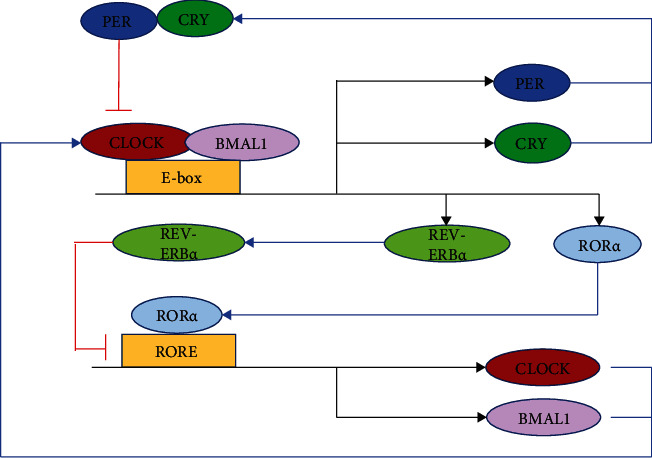
Molecular rhythm mechanism of the biological clock [22].

In human physiological activities, the biological clock determines the most basic physiological changes such as the sleep-wake cycle, respiration, blood pressure, heart rate, body temperature, and hormone secretion. Therefore, once the biological clock is disturbed, it will lead to mental illness and neurodegenerative diseases, infection, inflammation, cardiovascular disease, tumor, diabetes, and other diseases [22]. It is precisely because the human body's gene expression, cell metabolism, organ operation, and system control all operate regularly like a clock and play an important role in maintaining the health of the human body. Therefore, countless researchers invested a lot of precious time and energy to explore the molecular mechanism of its inner working mode, and finally, three American geneticists Jeffrey C. Hall, Michael Rosbash, and Michael W. Young revealed its specific molecular mechanism. The core of this circuit consists of bHLH and the PAS heterodimeric transcriptional activator (CLOCK or NPAS2 with BMAL1). In mammals, activating genes bind to E-box elements in the core circadian clock period (Per1, Per2, or Per3) and cryptochrome genes (Cry1 or Cry2) and then give negative feedback to control their transcription. Feedback timing is regulated by posttranscriptional modifications, especially posttranslational modifications. A common regulatory motif is rhythmic phosphorylation and rhythmic degradation of circadian clock components, which are usually accomplished through the ubiquitin-proteasome system. This core loop is enhanced by embedding other transcriptional feedback loops through the activation of Rev-erb*α* and Ror*α* by CLOCK-BMAL1. Other transcription factors provide feedback and regulate CLOCK activity, including USF1 and Dec1-Dec2. Studies in mice with disrupted core circadian clocks have shown that the rhythmicity of physiological processes results from the expression of oscillatory genes located downstream of this core transcriptional oscillator [22]. In general, the molecular mechanism network of the circadian clock is formed by the mutual control coupling of different genes under the influence of positive and negative feedback [54] (in Figure 2).

## 4. Biological Clock Gene Expression in IBD

The most extensive immunohistochemical analysis of IBD intestinal mucosal samples has found that the expression of five core circadian proteins (BMAL1, PER1, PER3, TIMELESS, and NAPS2) in the mucosal epithelium of IBD patients is reduced compared with controls. The expression of BMAL1 and PER1 was more significantly decreased in UC patients, the expression of PER3, TIMELESS, and NPAS2 was decreased in CD patients, and the expression of BMAL1 in mucosal inflammatory cells in IBD patients was decreased [27]. In another study, 29 patients with IBD (15 with CD and 14 with UC) were recruited, and mucosal biopsies from inflamed or adjacent noninflamed areas of the colon were used to assess IBD using genome-wide cDNA microarray analysis.

### 4.1. Circadian Gene Expression

The study examined a total of 150 circadian genes involved in pathways that control key cellular processes and tissue function. In CD specimens, 50 genes were differentially expressed, and 21 genes were upregulated compared with healthy colonic mucosa. In UC specimens, 50 genes were differentially expressed, and 27 genes were upregulated compared with healthy colonic mucosa. The core clock genes ARNTL2 and RORA were upregulated, while CSNK2B, NPAS2, PER1, and PER3 were downregulated in CD specimens. In contrast, ARNTL2, CRY1, CSNK1E, RORA, and TIPIN were upregulated in UC, whereas NR1D2 and PER3 were downregulated in UC. In conclusion, there are differences in circadian gene expression between normal and diseased intestinal mucosa in CD and UC patients [55]. This study reveals that dysregulated genes identified by transcriptome analysis in major IBD may play key roles in pathophysiological mechanisms and may suggest novel therapeutic approaches.

## 5. The Biological Clock Affects the Intestinal Barrier Function and Participates in the Pathogenesis of IBD

**Figure 3:**
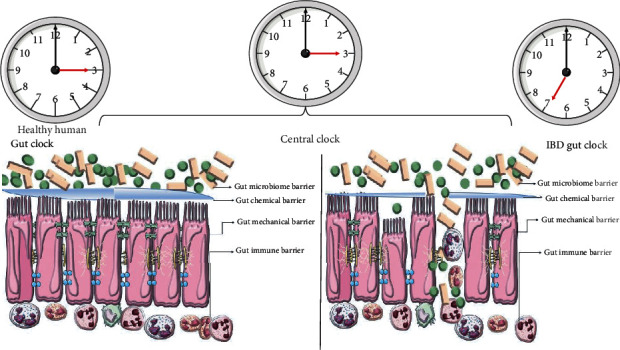
The effect of the biological clock on the intestinal barrier of healthy subjects and IBD.

The circadian clock also exists in the gut as a highly conserved and orchestrated molecular timer. Many digestive functions have daily rhythms, and circadian rhythms are associated with gut immune system function, gut microbes, mechanical barriers, and chemical barriers [32, 56–61]. Therefore, once the circadian rhythm is disturbed, it will affect the intestinal barrier function and induce IBD (Figure 3). The effects of the circadian clock on the above-mentioned four intestinal barrier functions are, respectively, introduced below, to clarify its role in the pathogenesis of IBD.

### 5.1. Biological Clock and Intestinal Mechanical Barrier

The effect of the biological clock on the intestinal mechanical barrier function is achieved through two aspects. On the one hand, the core clock gene Bmal1 of the biological clock can regulate the regeneration of intestinal epithelial cells by affecting cytokines, cell cycle, and cell proliferation, thereby causing daily variation in the self-renewal of intestinal epithelial cells [62]. In pathological conditions, the circadian clock operates in the intestinal epithelium, deletion of the core circadian clock gene BMAL1 disrupts the circadian clock and rhythmic proliferation of the intestinal epithelium, and circadian activity in the gut involves the rhythmic production of inflammatory cytokines and subsequent activation of protein kinase responses. Rhythmic activation of the stimulus-response pathway is reported in original research article written by Stokes et al. [62]. In addition, the circadian clock can also regulate the apoptosis of intestinal epithelial cells. Studies have demonstrated that the antiapoptotic genes Birc5 and Survivin are involved in the circadian regulation of cyclin-dependent kinase inhibitor toxicity in mouse colon cells [63]. Feeding rhythm is involved in the regulation of programmed cell death in the rat small intestine [64]. On the other hand, intestinal permeability is regulated by the biological clock. Among them, some scholars used real-time PCR to analyze wild-type mice and Period2 with a key clock gene every 4, 6, or 12 hours and found that the mRNA and protein expression levels of Occludin and Claudin-1 were in the colonic epithelium of wild-type mice, showing daily variation, while they were constitutively high in mPer2 (m/m) mice. Colonic permeability in wild-type mice showed daily changes that were inversely correlated with expression levels of Occludin and Claudin-1 proteins, whereas mPer2 (m/m) mice exhibited lower colonic permeability and increased sensitivity to glucosamine. Increased susceptibility to glycan sodium sulfate-induced colitis is reported in article written by Oh-Oka et al. [65]. It can be seen that maintaining the normal rhythm of the circadian clock is crucial for maintaining the integrity of the intestinal mechanical barrier. In the future, circadian clock therapy can be carried out to target and regulate the proliferation, repair, and apoptosis of intestinal epithelial cells and increase the expression level of tight junction proteins. Promoting the regeneration of intestinal epithelial cells in IBD and increasing the expression level of tight junction proteins can repair the dysfunctional intestinal mechanical mucosal barrier, which provides a promising treatment for IBD from the perspective of targeting the repair of intestinal mechanical barrier function.

### 5.2. Biological Clock and Intestinal Immune Barrier

A large number of studies have confirmed that there is a biological clock rhythm in the intestinal immune system, and the biological clock rhythm can affect the intestinal immune function [58, 59, 66, 67]. The effect of the biological clock on the intestinal immune barrier is achieved in two ways. First, the circadian clock can influence the function of the innate immune system in the gut. It is well known that group 3 innate lymphoid cells (ILC3s) are a cell group in the intestinal innate immune system, which are abundant in the lamina propria of the intestinal mucosa and are key regulators of intestinal inflammation. We found that intestinal ILC3s display circadian expression of clock genes and ILC3-related transcription factors and that ILC3-autonomous ablation of the circadian regulator Arntl leads to disruption of intestinal ILC3 homeostasis, impaired epithelial reactivity, dysbiosis, and susceptibility to intestinal infection. Increased sensitivity can easily lead to IBD [59]. It is well known that macrophages are key innate immune cell components in the pathogenesis of IBD, which are also controlled by circadian rhythms. In vitro experiments have confirmed that macrophages express typical clock genes such as Bmal1, Cry1-2, per1-3, and Rev-erb*α*, and in the case of lipopolysaccharide injection, macrophages express proinflammatory cytokines such as TNF-*α* and IL-6 that are also controlled by circadian rhythms [68]. Therefore, abnormal circadian rhythm leads to abnormal expression of proinflammatory factors in macrophages, which can lead to intestinal inflammation and tissue damage. Additionally, neutrophils, as effector innate immune cells of acute inflammation, have long been reported to play a role in maintaining intestinal homeostasis and IBD pathogenesis [69]. However, neutrophils are also affected by circadian rhythms. The study found that the superoxide-producing capacity of neutrophils also depends on the time of day. Consistent with this, the number of opsonized bacteria engulfed by neutrophils also showed time-dependent differences, with clearance of pathogens showing a daily rhythm [70]. Based on these changes, whether the circadian rhythm changes of neutrophils are closely related to the diagnosis and treatment of IBD and clinical prognosis, it will be worth further basic and clinical research to clarify the internal relationship between the two; second, the circadian clock can affect adaptive immune cells leading to IBD. It has been shown that an imbalance between T helper 17 (Th17) and regulatory T (Treg) cells differentiated from CD4+ T cells contributes to IBD. Th17 cells promote tissue inflammation, and Treg cells suppress autoimmune responses in IBD. Therefore, Th17/Treg cell balance is crucial [71]. However, abnormal circadian rhythms can lead to IBD by affecting factors that maintain their balance such as bile acid metabolism [72], intestinal flora homeostasis [57], and expression of cytokines [73]. Of course, the circadian clock can also influence other adaptive immune cells to cause IBD. In conclusion, circadian rhythms contribute to IBD by causing abnormalities in innate and adaptive immunity. Therefore, it is necessary to carry out real-world studies using immunosuppressive therapy combined with circadian clock therapy for some patients who are clinically ineffective with immunosuppressive therapy, which may greatly improve efficacy and reduce drug toxicity or adverse reactions. Confirmed in real-world studies, this is expected to be incorporated into clinical guidelines for the diagnosis and treatment of IBD, thus opening up a new mode of medication.

### 5.3. Biological Clock and Intestinal Microecological Barrier

It has been found that, in mice and humans, the gut microbiota exhibits circadian oscillations that are influenced by feeding rhythms, resulting in compositional and functional characteristics at specific times of the day [74], and disturbances in the circadian rhythm can affect the gut. The composition and function of the microbiota affect human health [32, 57, 60, 75–79] and even lead to the occurrence of IBD [30, 80, 81]. This is mainly due to circadian rhythm disturbances affecting the composition of the gut microbiota [82] and circadian disruption of the host, altering the composition of bacterial populations in the gut [74]. It was found that a decrease in anti-inflammatory bacteria and an increase in proinflammatory bacteria were observed in IBD patients compared with healthy individuals [83], with a decrease in gut microbiota diversity and a decrease in the abundance of *Firmicutes* [84, 85]. For example, the first CD-associated *E. coli* with proinflammatory properties isolated from adult CD patients was the adherent-invasive *E. coli* [84]. Increased numbers of adherent-invasive *E. coli* have been reported in approximately 38% of patients with active CD, compared with 6% in healthy subjects [86]. The increase of pathogenic bacteria with the ability to adhere to the intestinal epithelium affects intestinal permeability, changes the diversity and composition of intestinal flora, and induces an inflammatory response by regulating the expression of inflammatory genes, thereby inducing intestinal inflammation [87]. In response to the imbalance of anti- and proinflammatory microbiota in IBD, there has been strong interest in the possible benefits of clinically modulated interventions with microbial agents (e.g., probiotics, prebiotics, antibiotics, and fecal microbiota transplantation) in the treatment of IBD [50], and certain results were achieved. However, if a better therapeutic effect can be achieved by adding microbial preparations based on circadian rhythm, it is worthy of further research to design randomized controlled experiments in the future.

### 5.4. Biological Clock and Intestinal Chemical Barrier

Studies have confirmed that circadian rhythms can regulate the expression levels of various intestinal enzymes [88], bile acids [72, 89, 90], and mucins [30]. It is well known that normal human bile acids can be divided into free bile acids and conjugated bile acids. The former includes cholic acid, deoxycholic acid, chenodeoxycholic acid, and a small amount of lithocholic acid, and the latter includes glycocholic acid, glycochenodeoxycholic acid, taurocholic acid, and taurochenodeoxycholic acid which are essential for the absorption, transportation, and metabolism of intestinal dietary fat and fat-soluble vitamins. In addition to the normal physiological functions described above, different types of bile acids play an anti-inflammatory role in IBD. Studies have found that the secondary bile acid ursodeoxycholic acid attenuates the release of proinflammatory cytokines (TNF-*α*, IL-6, IL-1*β*, and IFN-*γ*) in colonic epithelial cells in vitro and prevents colonic inflammation in vivo development [91]. Therefore, the secondary bile acid ursodeoxycholic acid may be a potential therapeutic target for IBD. In addition, taurocholate was able to reduce the active accumulation of MPO and the levels of IL-1*β*, IFN-*γ*, and TNF-*α* in colon tissue in TNBS-induced ulcerative colitis in mice, thereby exerting anti-inflammatory effects [92]. However, key enzymes in bile acid synthesis and activation by bile acids and nuclear receptors involved in bile acid regulation showed marked circadian changes. Once the circadian rhythm is dysregulated (interruption of the circadian rhythm, feeding restriction, and sleep disruption), bile acid homeostasis is disrupted, thereby releasing inflammatory factors leading to the development of IBD [89]. Based on the results of the above-mentioned existing studies, thinking about how to use the circadian rhythm to maintain the homeostasis of bile acid metabolism, letting the body's metabolite bile acid play an anti-inflammatory effect, and then managing IBD is the most economical and safest treatment strategy, which is worth designing In-depth research on basic and clinical experiments is carried out to develop a new regimen of maintaining bile acid metabolism homeostasis combined with circadian clock therapy for the treatment of IBD.

## 6. Clinical Significance and Potential Therapeutic Approaches of Clock Genes

In a prospective study of 32 IBD patients (8-21 years old) and 18 healthy individuals, the expression levels of clock genes (CLOCK, BMAL1, CRY1, CRY2, PER1, and PER2) were analyzed in the peripheral blood and intestinal mucosa samples, and the expression levels of clock genes (CLOCK, CRY1, CRY2, PER1, and PER2) in the inflamed intestinal mucosa of the patients were significantly lower than those of the control intestinal mucosa (*P* < 0.05). Compared with the control group, the expression levels of all clock genes except PER2 were also significantly decreased in the noninflammatory intestinal mucosa of the patients (*P* < 0.05). The expression levels of clock genes (CLOCK, BMAL1, CRY1, CRY2, PER1, and PER2) were lower in leukocytes of IBD patients compared with controls, suggesting that clock gene disruption is an initial manifestation of IBD [93]. Therefore, we can clinically detect changes in clock gene expression in patients with inflamed intestinal mucosa samples and leukocytes and then intervene in the disease early. In addition, studies have found that Rev-erb*α* can inactivate the Nlrp3 inflammasome, and activation of Rev-erb*α* can effectively relieve colitis. Therefore, Rev-erb*α* is expected to be a new drug target for the prevention and control of colitis [94]. In addition, in clinical samples from colitis-associated colorectal cancer patients, low expression of the Bmal1 gene in paracancerous tissues and tumor central regions was closely associated with a poorer prognosis in colitis-associated colorectal cancer patients [95]. This study suggests that in the management of colitis-related colorectal cancer in the future, by detecting the Bmal1 gene in the adjacent tissue and the central tumor tissue, the clinical prognosis of patients can be assessed, and a theoretical basis for further clinical diagnosis and treatment can be provided. According to the above studies, clock genes have some functions as shown in Table 1 in the management of IBD. However, the value of the role of clock genes in IBD will be more discovered in future research, so as to better manage IBD patients.

## 7. Summary and Outlook

This review systematically summarizes the composition and function of the intestinal barrier, the molecular mechanism of the biological clock, and how the biological clock participates in the pathogenesis of IBD by affecting the function of the intestinal barrier. It is hoped that the potential value of the biological clock in the management of IBD will be paid attention to, to better serve the patients. Finally, we always have reason to believe that after three American scientists were awarded the Nobel Prize in Physiology or Medicine in 2017 for discovering the molecular mechanism of the biological clock [96], coupled with single-cell sequencing [97], various omics (genomics [98], proteomics [99], transcriptomics [100], metabolomics [101], etc.), big data [102], 5G technology [103], and artificial intelligence [104] which are widely used in the medical field and development, the application value of biological clock in medicine will be deeply excavated, especially the research on using the biological clock to manage IBD will open a new era, and there will be more research to reveal the role of the biological clock in IBD from the whole, tissue, organ, cell, gene, and molecular level. The mysterious veil of pathogenesis has resulted in many new means to accurately prevent the occurrence of IBD based on the principle of the biological clock and is expected to greatly improve the treatment effect of IBD patients, thereby benefiting the majority of patients.

## Figures and Tables

**Table 1:** Potential clinical value of clock genes for management of IBD.

Clinical value	Method
Early prediction of the occurrence of IBD	Analysis of clock gene expression levels in peripheral blood and intestinal mucosa samples [93]
Finding new drug targets for treating IBD	Rev-erb*α* is expected to be a drug target for the prevention and control of colitis [94]
Assessing the prognosis of colitis-related colorectal cancer	Detection of Bmal1 gene in paracancerous tissue and central tumor tissue [95]
